# Tryptophan Metabolism in Neurodevelopment and Its Implications For Neurodevelopmental Disorders

**DOI:** 10.1007/s12035-025-05437-9

**Published:** 2025-11-20

**Authors:** Maria Grazia Giuliano, Paola Tognini

**Affiliations:** Health Science Interdisciplinary Center, School of Advanced Studies, Pisa, Italy

**Keywords:** Tryptophan, Kynurenine, Indole, Gut microbiota, Neurodevelopment, Neurodevelopmental disorders

## Abstract

The role of tryptophan metabolism has been recognized in a wide range of physiological and pathological processes but is still only partially understood. Growing evidence highlights the importance of maintaining tryptophan homeostasis throughout life, with its disruption now linked to various neuropsychiatric conditions spanning from early life to aging. While it is increasingly evident that alterations in tryptophan metabolism have significant implications for both neurodevelopmental and neurodegenerative disorders, research has predominantly focused on the latter, leaving neurodevelopmental aspects comparatively underexplored. This review provides a comprehensive overview of both preclinical and clinical studies, highlighting the intricate relationship between tryptophan metabolism and neurodevelopment. Particular focus is given to the kynurenine pathway and gut microbiota-derived indole production, two interconnected metabolic branches with profound effects on brain maturation, plasticity, and immune regulation. Finally, we examine the pathophysiological consequences of tryptophan dysregulation in neurodevelopmental disorders, including autism spectrum disorder, attention-deficit/hyperactivity disorder, and Rett syndrome. We also discuss potential therapeutic strategies targeting tryptophan metabolism in these conditions.

## Introduction: Tryptophan Metabolism as a Key Source of Neuroactive Compounds Regulated by Endogenous and Exogenous Factors

**Fig. 1:**
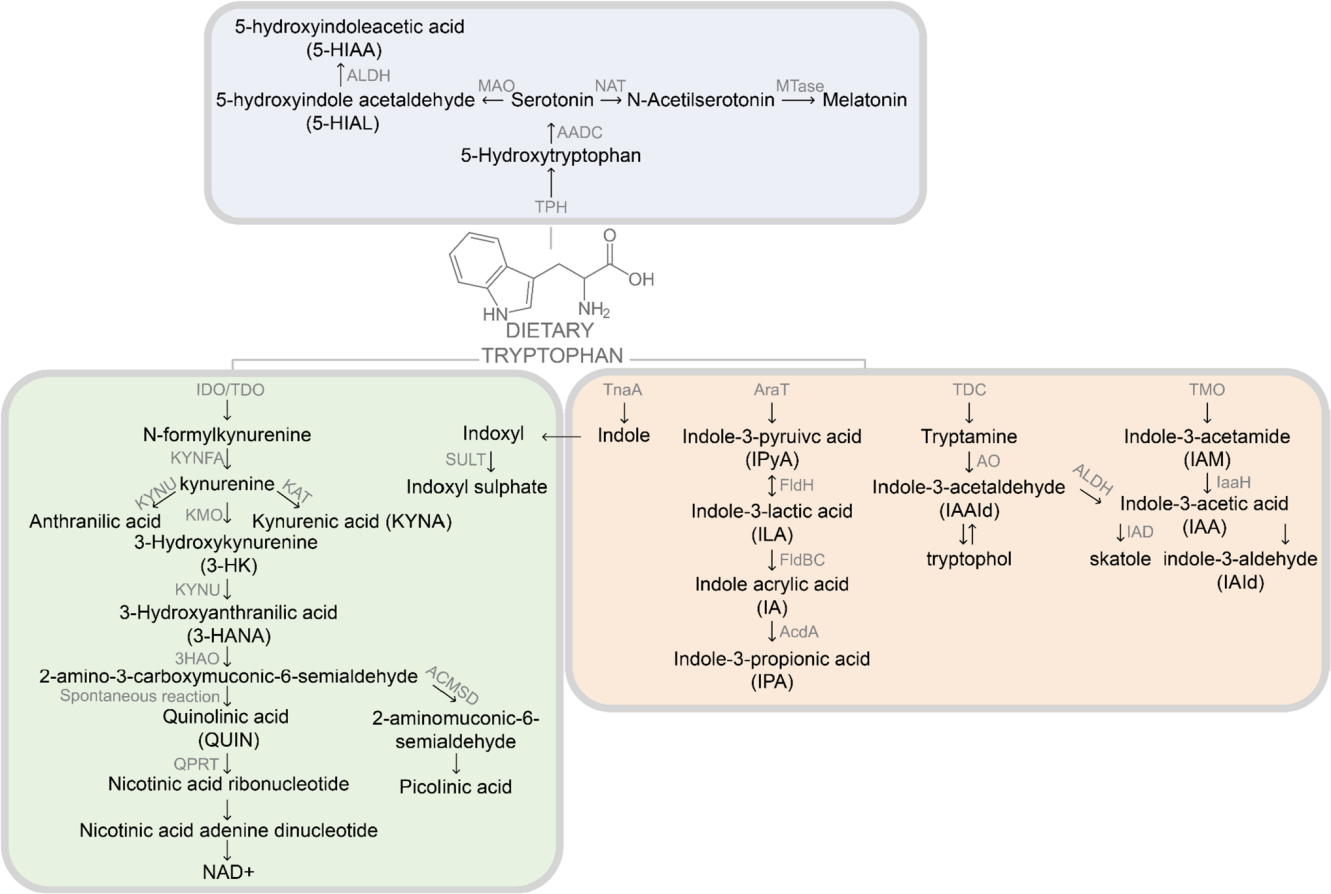
Overview of major tryptophan metabolic pathways. Tryptophan (Trp) is metabolized via three major routes: the serotonin/melatonin pathway (top, blue box), the kynurenine pathway (left, green box), and the microbial indole pathway (right, orange box). Key enzymes are indicated along each pathway. TPH, tryptophan hydroxylase; AADC, aromatic L-amino acid decarboxylase; NAT, aralkylamine N-acetyltransferase; MTase, methyltransferase; MAO, monoamine oxidase; ALDH, aldehyde dehydrogenase; IDO, indoleamine 2,3-dioxygenase; TDO, tryptophan 2,3-dioxygenase; KMO, kynurenine 3-monooxygenase; KYNU, kynureninase; KAT, kynurenine aminotransferase; QPRT, quinolinate phosphoribosyltransferase; ACMSD, aminocarboxymuconate semialdehyde decarboxylase; TnaA, tryptophanase; SULT, sulfotransferase; AraT, aromatic amino acid transaminase; FldH/FldBC, aldehyde and dehydrogenase enzymes; AcdA, acyl-CoA dehydrogenase; TDC, tryptophan decarboxylase; AO, amine oxidase; TMO, tryptophan monooxygenase; IAD, indole-3-acetate decarboxylase; IaaH, indole-3-acetamide hydrolase; 5-HT, serotonin; 5-HIAA, 5-hydroxyindoleacetic acid; 5-HIAL, 5-hydroxyindole acetaldehyde; IPA, indole-3-propionic acid; IAA, indole-3-acetic acid; ILA, indole-3-lactic acid; IAAlD, indole-3-acetaldehyde; IPyA, indole-3-pyruvic acid; IAld, indole-3-aldehyde; QUIN, quinolinic acid; KYNA, kynurenic acid; NAD⁺, nicotinamide adenine dinucleotide

L-Tryptophan (Trp), the least abundant amino acid in the human body, contains an indole ring that animals cannot synthesize, which must be obtained through diet or protein degradation [1]. Once released from dietary proteins, Trp is converted into indole by several phyla of gut bacteria, producing neuroactive indole derivatives that influence brain function through a partially unknown mechanism [2–5]. The remaining Trp serves as a precursor of serotonin (5-hydroxytryptamine, 5-HT), a key neurotransmitter/neuromodulator within the enteric and central nervous systems (CNS), primarily produced in gut enterochromaffin cells [6, 7]. Alternatively, Trp and indoles are absorbed through the intestinal epithelium and delivered to the hepatic portal system [8]. In the liver, indole is oxidized and sulphated to indoxyl sulfate, a neurotoxic compound that, if in excess, can cause the accumulation of neurotransmitters which block the efflux transporter in the blood-brain barrier (BBB) [9]. Only 5% of Trp that reaches the liver is used for protein synthesis, while the other 95% follows the kynurenine pathway (KP), producing a range of neuroactive intermediates collectively known as kynurenines (Fig. 1).

**Fig. 2:**
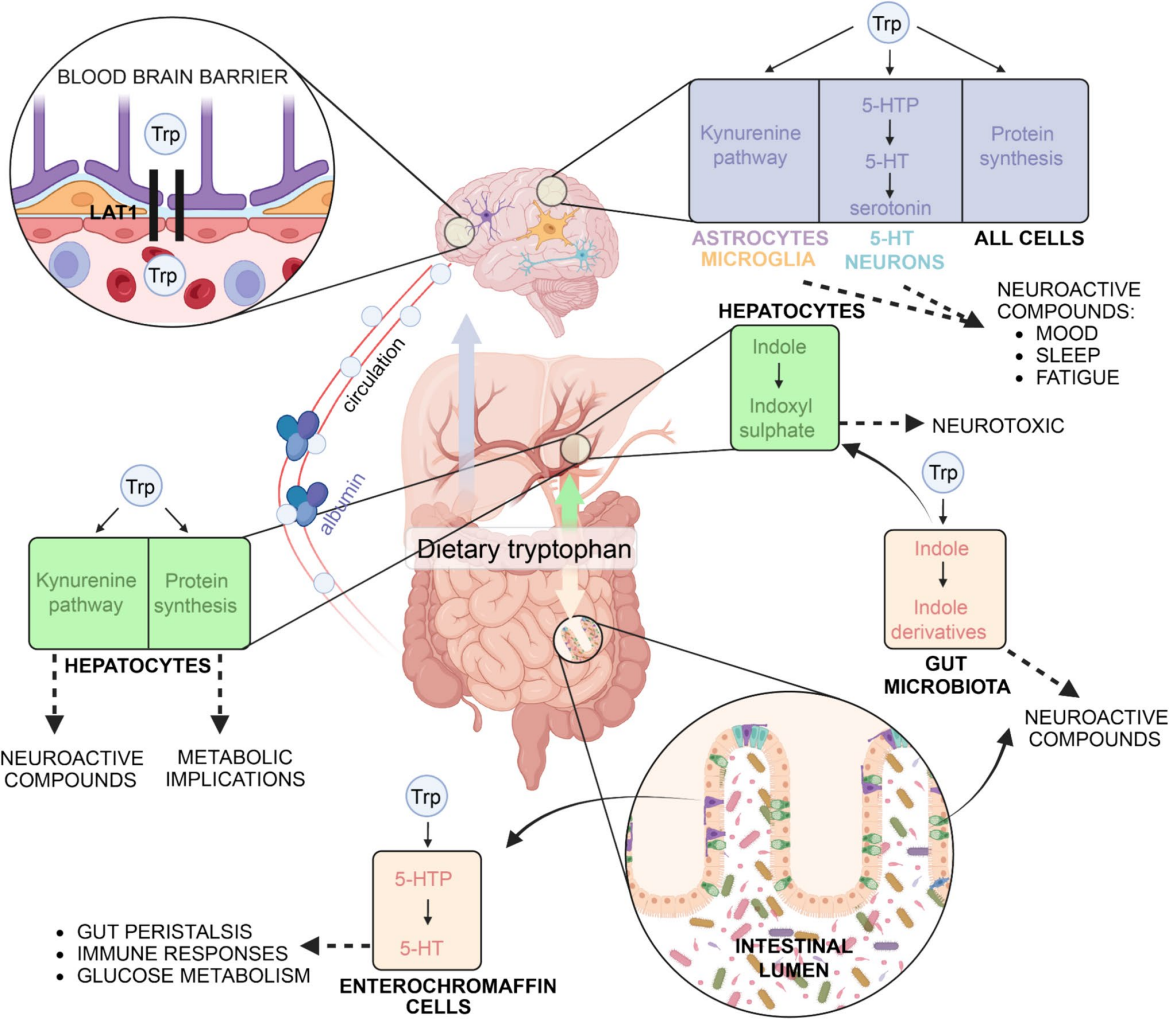
Tryptophan distribution and metabolic fate across tissues. Schematic illustration of dietary tryptophan (Trp) uptake and its major metabolic pathways in different tissues, including the serotonin pathway (gut enterochromaffin cells and brain), the kynurenine pathway (liver), and microbiota-mediated indole pathway (gut microbiota, liver)**.** Briefly, Trp is transported across the blood–brain barrier via LAT1 (L-type amino acid transporter 1) and converted into serotonin (5-HT) through the intermediate 5-hydroxytryptophan (5-HTP). In peripheral tissues, Trp is largely catabolized via the kynurenine pathway, generating various neuroactive and immunomodulatory metabolites. In the gut, microbial enzymes convert Trp into a wide range of indole derivatives. Solid black arrows represent enzymatic steps or metabolic intermediates within each pathway. Dashed lines indicate physiological roles or downstream effects of Trp-derived metabolites. Trp, tryptophan; 5-HTP, 5-hydroxytryptophan; 5-HT, serotonin (5-hydroxytryptamine); LAT1, L-type amino acid transporter 1. Illustration created with BioRender.com.

These intermediates have been shown to influence brain function, with imbalances linked to various neurological disorders such as schizophrenia, depression, Alzheimer’s disease, and epilepsy [4, 10–16]. Unmetabolized Trp enters the bloodstream, either albumin-bound or free, with the latter crossing the BBB via L-type amino acid transporters (LAT) [17]. In the brain, Trp undergoes metabolism for (i) kynurenines, (ii) nicotinamide adenine dinucleotide (NAD^+^), (iii) 5-HT (10–20%), and (iv) melatonin [18]. Together, these biochemical cascades influence mood regulation, stress responses, sleep, immune function, and overall neurological health [19–21] (Fig. 2). Trp deficiency impairs protein synthesis, causing muscle and weight loss [18]. Due to its neuroactive properties, imbalanced intake also affects the CNS, influencing mood, fatigue, and perception [22, 23]. In the Western world, excessive Trp consumption (70–200 mg/kg) is common, often through diet or supplementation with antidepressants that augment 5-HT function [24]. Such high levels of Trp intake are associated with a variety of side effects, from mild symptoms like tremor, nausea, and dizziness, to rare cases of serotonin syndrome—a severe condition marked by delirium, myoclonus, hyperthermia, and coma, requiring urgent medical care [25]. A high-fat diet is also known to negatively affect Trp metabolism through multiple mechanisms. These include immune activation, which leads to elevated cytokine levels and the accumulation of neurotoxic kynurenine metabolites [26]; increased activity of indoleamine 2,3-dioxygenase (IDO), the first and rate-limiting enzyme of the KP, which shifts Trp metabolism toward the kynurenine branch and may trigger inflammatory responses [27]; and modulation of the gut microbiota, with a reduction in beneficial bacterial species and an increase in Trp degradation [28]. Several pieces of evidence also indicate that fiber intake is strongly connected to Trp metabolic fate, with a central role played by the intestinal microbiota [29–32].

Trp metabolism plays a critical but incompletely understood role in health and disease. While the 5-HT/melatonin pathway is well characterized, the KP and microbiota-derived indoles have only recently gained attention [33, 34]. Emerging evidence links imbalances in these pathways to pathology, highlighting their therapeutic potential. This review examines the impact of kynurenine and indole metabolites on neurodevelopment, particularly neural function and neuroinflammation, and evaluates preclinical and clinical findings linking Trp metabolism to CNS health. Finally, we discuss whether targeting these pathways could offer new therapeutic strategies for neurodevelopmental disorders.

### Tryptophan Metabolism in the CNS

Kynurenines have relevant and unique effects in the CNS, and evidence suggests that they play an important role in brain development [35]. L-kynurenine (kynurenine) is metabolized from Trp by the IDO1 and IDO2 enzymes and Trp 2,3-dioxygenase (TDO2), producing N-formylkynurenine, which rapidly converts to kynurenine. Kynurenine enters the brain from circulation and is taken up by astrocytes and microglia. In astrocytes, kynurenine aminotransferase (KAT) catalyzes the irreversible transamination of kynurenine to kynurenic acid (KYNA) [36], a neuroactive antagonist of α7 nicotinic acetylcholine (α7nACh) and *N*-methyl-D-aspartate (NMDA) receptors [37]. In microglial cells [38], the kynurenine 3-monooxygenase (KMO) metabolizes kynurenine to 3-hydroxykynurenine (3-HK), leading to 3-hydroxyanthranilic acid via kynureninase catalysis followed by quinolinic acid (QA), a neurotoxic NMDA receptor agonist that promotes free radical generation [37]. KYNA, in contrast, reduces excitotoxicity by antagonizing ionotropic glutamate receptors [39]. Approximately half of brain kynurenine is derived peripherally, linking gut and liver Trp metabolism to kynurenine levels in the CNS [37].

Trp metabolites also activate the Aryl hydrocarbon receptor (AhR), a transcription factor influenced by environmental, microbial, and metabolic signals, with key roles in metabolism, cell differentiation, intestinal barrier integrity, and immune function [40–44]. In the CNS, AhR activation in brain microvessels and the BBB, where its expression is elevated, disrupts vascular homeostasis, contributing to neurodegenerative diseases [45]. This disruption is further exacerbated by oxidative stress [46], activation of inflammatory pathways, induction of endothelial cell senescence, and vascular calcification, as seen in Alzheimer’s-related cerebral amyloid angiopathy. Dysregulated AhR signaling also affects circadian rhythms, while gut microbiota dysbiosis may amplify brain pathology via Trp-AhR pathways [45, 47]. Moreover, kynurenine-mediated AhR activation is implicated in ischemic injury but may have neuroprotective effects in retinal ischemia/reperfusion injury [48]. During postnatal development, excessive AhR activation has been shown to disrupt olfactory interneuron migration and dendritic growth in mice [49, 50]. Also, AhR modulation appears essential for normal brain maturation in zebrafish embryos supporting its relevance during prenatal and early postnatal brain development. As many AhR-modulating metabolites derive from Trp, this pathway likely links immune signaling to neurodevelopment, with AhR activity potentially helping to explain the role of Trp in both brain maturation and disorder vulnerability.

Finally, the accumulation of kynurenine intermediates like QA in the CNS is also linked to Huntington’s disease, schizophrenia, and other inflammatory conditions [51]. Furthermore, 3-hydroxy-anthranilic acid and 3-hydroxy-kynurenine can induce neuronal apoptosis or necrosis, contributing to neurodegeneration.

Dysregulated KP may impact postnatal neurodevelopment by interfering with the balance between neurotoxic (QA) and neuroprotective (KYNA) metabolites in response to inflammation. Given its role in inflammation, excitatory/inhibitory balance, and neuronal viability, KP could be a promising therapeutic target for early-life neurological conditions.

## Crucial Role of Tryptophan Metabolites in Neurodevelopment

### Prenatal Stages of Brain Development

Dietary Trp is converted into neuroactive molecules that influence neuronal and glial function throughout life. Its importance during gestation is well established [52–54], as maternal circulating Trp supports fetal brain development by (i) contributing to protein synthesis and cell membrane formation [55], (ii) shaping the serotonergic system which impacts anxiety and depression [56], and (iii) regulating the kynurenine-cytokine pathway. Disruptions in the Trp/kynurenine ratio can predispose the fetus to neuropsychiatric conditions [57–60] and play a role in fetal immune development and rejection. [61, 62] Maternal Trp serves as a key substrate for placental kynurenine metabolism, with KP enzyme activity detected as early as the first trimester [63]. Placental Trp metabolism, a relevant player in the so-called placenta-brain axis [64], shifts throughout pregnancy, favoring 5-HT synthesis in early gestation and kynurenine production near term [52]. Notably, tight regulation of maternal 5-HT levels during the first trimester is essential. This is supported by a recent observational study involving 1115 women, which found that higher concentrations of 5-HT were associated with reduced embryonic and fetal growth, as well as an increased risk of small-for-gestational-age infants. Interestingly, this adverse outcome appears to be mitigated by elevated KYN levels, highlighting the importance of balanced Trp metabolism for healthy fetal development [65]. In line with these clinical findings, excess maternal Trp intake has been shown to cause fetal brain hyperserotonemia in rats, disrupting serotonergic system development and GH-IGF-1 signaling, thus impairing general growth [65]. Also, Trp metabolites possess antioxidant properties, suggesting a role in managing oxidative stress during pregnancy. Decreased fetal body weight as a consequence of excess Trp during gestation has also been observed in pregnant mice fed with a high L-Trp diet (L-Trp intake: 7.0 g/kg BW/day) [66].

Finally, since Trp metabolites exhibit antioxidant properties [67], the regulation of Trp metabolism could be used to control/attenuate oxidative stress during abnormal pregnancy.

During pregnancy, maternal Trp imbalance, influenced by stress and/or dietary factors, may disrupt serotonergic pathways and KP, affecting not only placenta metabolism and fetal growth, but also offspring behavior later in life. Disruption of these pathways is believed to contribute to the emotional and behavioral dysfunction often observed in individuals exposed to prenatal stress [61, 62, 68, 69]. Indeed, prenatal stress leads to sex-specific behavioral changes in mice: females show depressive-like traits along with decreased hippocampal 5-HT levels and an increased 5-HT turnover rate in both the hippocampus and brainstem, while males exhibit anxiety-like behavior with elevated QA [69]. Additionally, studies in pregnant mice have shown that acute stress leads to a transient increase in fetal brain levels of the neuroprotective KYNA, while levels of the neurotoxic metabolites 3-hydroxykynurenine and QA remain unchanged [61, 62, 68]. KYN administration during early pregnancy also induces long-term behavioral changes in the offspring [70], further underscoring the critical role of Trp metabolism in neurodevelopment.

In addition, Trp metabolism may link maternal inflammation to fetal brain development, with diet and stress shaping the serotonergic–kynurenine balance and influencing long-term neurodevelopment. For instance, mid-pregnancy inflammation in a mouse model exposed to moderate doses of viral polyinosinic:polycytidylic acid (poly I:C) [71, 72] has been reported to upregulate Trp conversion to 5-HT in the placenta, leading to accumulation of placenta-derived 5-HT and blunted 5-HT axonal outgrowth specifically in the fetal forebrain [73]. Conversely, an ex vivo study on human placenta has recently shown that exposure to bacterial LPS and viral poly I:C impairs Trp homeostasis, resulting in decreased production of 5-HT and an imbalanced QA/KYNA ratio [74]. This is in line with previous evidence indicating that an inflammatory placental environment in humans is associated with upregulation of IDO1, indicating increased flux through the kynurenine branch, and concomitant downregulation of the 5-HT branch [75]. These conflicting findings highlight discrepancies in studies on placental inflammation and Trp metabolism in the placenta and fetus. While some stem from methodological differences, the system’s complexity, involving metabolizing enzymes and protein transporters, makes it difficult to pinpoint exact mechanisms.

During neurodevelopment, Trp metabolism may mediate the complex gut-brain-immune crosstalk. The gut microbiota, shaped by maternal diet, stress, and inflammation, could influence the availability and fate of Trp, including its conversion into neuroactive and immunomodulatory metabolites [76–78]. These Trp-derived metabolites, in turn, may act as signaling molecules along the gut-brain axis. Embryonic Trp exposure in chicken embryos has been shown to reduce body weight and aggressive behavior in male offspring, while also altering gut microbiota composition and function, supporting the hypothesis that Trp excess during gestation leads to long-term metabolic and behavioral changes via microbiota-dependent mechanisms [52]. Chen et al. (2020) demonstrated that prenatal stress elevates placental Trp and 5-HT levels and causes long-term behavioral deficits in the offspring. These effects were absent in germ-free (GF) mice, implicating Trp-metabolizing bacteria such as *Parasutterella* and *Bifidobacterium* as key modulators of these outcomes [79]. Supplementation of stressed dams with *Bifidobacterium dentium* mitigated maternal and fetal inflammation, restored levels of neuroprotective Trp metabolites (indole-3-propionic acid (IPA), KYNA), and improved offspring social behavior [80]. Furthermore, fecal microbial transfer from Western diet-fed obese dams to chow-fed GF lactating dams suppressed circulating Trp-derived AhR ligands (e.g., indole, indole-3-acetate) and impaired innate immune responses in the offspring [81]. These findings underscore the potential role of Trp metabolites, shaped by the maternal microbiome, in modulating fetal immune and neurodevelopmental trajectories.

### Postnatal Stages of Brain Development

While evidence has demonstrated that fetal exposure to altered Trp metabolism affects brain development, the postnatal period is also a critical window, requiring tight regulation of Trp pathways for healthy brain maturation. Neonatal infections with a neurotropic influenza A virus can trigger the KP, leading to a consistent increase in the expression of IDO. This activation is also marked by a temporary rise in KYNA levels in the brains of infected mice. In genetically susceptible mice, this early-life KP activation can result in long-term deficits in sensorimotor gating [82]. Lead exposure during the lactation period leads to increased levels of both KYNA and 3-HK, along with elevated KMO activity, immediately following the exposure period. The elevated KYNA levels were found to persist into adulthood and correlated with cognitive impairments [83]. Similarly, maternal deprivation in rats alters the expression of IDO at different developmental stages. Specifically, IDO expression decreases in the hippocampus during infancy but increases in the prefrontal cortex in adulthood. This model of early-life stress also leads to depressive-like behaviors in adult rats [84], suggesting that the timing of KP alterations is critical. Finally, when the KYNA precursor, kynurenine, is administered to rat dams from gestation through the early postnatal period, it results in elevated brain KYNA levels in the offspring. These animals exhibit cognitive deficits in adulthood [85], suggesting that increased brain KYNA during a critical developmental window may have lasting negative consequences on cognition.

Overall, the current literature highlights how multiple factors, including maternal diet, psychological stress, gut health and inflammation—all deeply interconnected and capable of influencing each other—may impact Trp metabolism homeostasis, with direct consequences on fetal brain development and long-term repercussions later in life [86]. Despite the complexity of Trp metabolism and its many derivatives, this opens promising therapeutic opportunities that could potentially be implemented during prenatal life.

## Consequences of Disrupted Tryptophan Metabolism: Foundations for Neurodevelopmental Disorders

Trp metabolites are critical for brain development, and their dysregulation may contribute to neurodevelopmental disorders, making this a relevant area for clinical research (Table 1). The KP, regulated by the inflammation-sensitive enzyme IDO, links Trp metabolism to immune function. Chronic low-grade inflammation is increasingly recognized as a factor in neurodevelopmental disorders [100] highlighting the importance of Trp metabolism in neuroimmune interactions.

**Table 1:** Alterations in tryptophan-derived metabolites across neurodevelopmental disorders

Disorder	Altered Trp metabolite	Tissue	Preclinical evidence	Clinical evidence	Ref
ASD	↓ Serotonin (5-HT)	Urine	-	✔	[9]
	↑ Serotonin (5-HT)	Whole blood, serum, platelets, brain	✔	✔	[87–89]
	↓ 5-Hydroxyindoleacetic acid (5-HIAA)	Urine	-	✔	[9]
	↑ 5-Hydroxyindoleacetic acid (5-HIAA)	Platelet	-	✔	[88]
	↑ Xanthurenic acid (XA)	Urine	-	✔	[9]
	↑ Quinolinic acid (QUIN)	Urine	-	✔	[9]
	↓ Kynurenic acid (KYNA)	Urine	-	✔	[9]
	↑ Indolyl 3-acetic acid (IAA)	Urine	-	✔	[9] 90]
	↑ Indolyl lactate (ILA)	Urine	-	✔	[9]
	↑ Indole	Feces	-	✔	[90]
	↑ Indole pyruvate (IPy)	Serum	✔	-	[89]
	↑ 3-Methylindole	Feces	-	✔	[90]
ADHD	↑ Tryptophan (Trp)	Blood	-	✔	[91, 92]
	↑ Kynurenine (KYN)	Blood, serum	✔	✔	[91–93]
	↑ Anthranilic acid	Serum, brain	✔	-	[93]
	↓ Kynurenic acid (human) (KYNA)	Blood	✔	✔	[91, 92]
	↑ 3-Hydroxyknurenine (mouse) ↓ 3-Hydroxyknurenine (human) (3-HK)	Serum, brain	✔	✔	[91–94]
	↓ 3‑Hydroxyanthranilic acid (3-HANA)	Serum	-	✔	[95]
RTT	↑ Kynurenine (KYN)	Brain	-	✔	[96]
	↓ Serotonin (5-HT)	Brain	✔	✔	[96, 97]
	↓ 5-Hydroxyindoleacetic acid (5-HIAA)	Brain	-	✔	[96]
	↓ Kynurenine (KYN)	Plasma	-	✔	[98]
	↓ Indole propionate (IPA)	Plasma	-	✔	[98]
	↓ Indole lactate (ILA)	Plasma	-	✔	[98]
MeCP2 duplication syndrome	↑ Serotonin (5-HT)	Serum	✔	-	[99]
	↓ Serotonin (5-HT)	Urine, cecum	✔	-	[99]
	↓ Tryptamine	Plasma	✔	-	[99]
	↓ 4-Hydroxyindole	Plasma	✔	-	[99]
	↓ 3-Indole propionate (IPA)	Plasma	✔	-	[99]
	↓ Indole lactate (ILA),	Plasma	✔	-	[99]
	↓ 5-Hydroxyindoleacetic acid (5-HIAA)	Plasma	✔	-	[99]

Furthermore, preclinical and clinical evidence links the KP to epilepsy, largely due to the properties of KYNA and QA. QA administration induces seizures in rodents [101, 102], and epilepsy-prone mice show increased QA and KP enzyme expression [103], while KYNA influences excitatory/inhibitory balance through glutamate receptors and calcium channel modulation [104]. Microbiota manipulation via antibiotics or IDO1 inhibition reduces seizures in a mouse model of infantile spasms by increasing hippocampal KYNA [105]. These findings suggest that early disruptions in KP metabolism may alter neurodevelopment and heighten susceptibility to epilepsy. Patients with epileptic spasms show reduced KYNA and KYNA/kynurenine ratio in the cerebrospinal fluid [15], while those with status epilepticus exhibit a significant Trp-KP alteration, leading to QA overproduction [106]. Additionally, the KP’s first and rate-limiting enzyme, IDO, found in microglia, astrocytes, neurons, and macrophages, is upregulated by inflammatory stimuli, including cytokines, LPS, amyloid peptides, HIV proteins, and interferon gamma [107].

The following section focuses on autism spectrum disorder (ASD) and attention deficit hyperactivity disorder (ADHD), two of the most prevalent and comorbid childhood neurodevelopmental disorders [108–110]. Both have been increasingly linked to immune dysfunction, gut-brain axis alterations, and metabolic abnormalities, making them relevant for exploring Trp metabolism in the context of neurodevelopment [111–113]. Although ASD and ADHD share phenotypic overlap and comorbidity, comparing their Trp metabolic profiles is challenging due to differences in study design, sample types, and metabolites analyzed [114]. Moreover, many studies do not directly link altered Trp levels to clinical features [9, 115, 116]. Still, both conditions appear to involve shifts in Trp metabolism, marked by serotonergic and KP imbalances, often linked to immune and gut microbiota changes [117].

Rett syndrome (RTT) is also discussed to offer insight into Trp-related pathways in a genetic disorder [98]. Although evidence is limited, this condition highlights the potential link between Trp metabolism and monogenic neurodevelopmental disorders, encouraging its investigation in other diverse neurodevelopmental pathologies.

### Autism Spectrum Disorders

Hyperserotonemia affects approximately one-third of individuals with ASD, who show elevated platelet and urinary levels of 5-HT and its metabolite 5-HIAA, compared to neurotypical individuals and those with intellectual disabilities outside the autism spectrum [9, 87, 88]. This peripheral increase is thought to result from heightened activity of enterochromaffin cells—the primary source of systemic serotonin. Notably, enterochromaffin cell hyperactivity has been observed in inflammatory conditions such as IBS and Crohn’s disease [118, 119]. Furthermore, inducing colitis in animal models similarly elevates both 5-HT levels and enterochromaffin cell counts [120, 121]. While these findings suggest a possible link between gut inflammation and hyperserotonemia in ASD, it is also plausible that both phenomena reflect a shared underlying immune alteration associated with ASD. Peripheral hyperserotonemia may not directly reflect central serotonergic activity, although it may still contribute to systemic immune signaling relevant to ASD pathophysiology, considering the multiple ways in which peripheral immune signals can enter the brain [122–126].

Alterations in serotonergic signaling have long been known to profoundly affect behavior in animal models. In line with this, knockouts of the 5-HT2B receptor in *Drosophila*—a model for studying social behavior abnormalities—induce autism-like phenotypes, including increased social spacing and repetitive grooming behaviors [127]. In the BTBR mouse model of ASD, overexpression of the 5-HT1A receptor reduces stereotyped behavior in the marble-burying test and increases time spent in the center during the open field test, suggesting a potential role in modulating both repetitive behaviors and anxiety-related features associated with ASD [128]*.*

Beyond 5-HT, urine metabolome analyses show altered KP metabolism in ASD, with a preference for xanthurenic acid and QA over neuroprotective KYNA, which may reflect immune activation. However, the specific tissue origin of these changes (e.g., brain, gut, or peripheral immune system) remains uncertain [9, 127]. A study on Egyptian children found reduced mRNA expression of enzymes involved not only in 5-HT catabolism (MAO), but also in KYNA production (AADAT) and QA production (HAAO) in blood samples from children with ASD, compared to those with learning disabilities and healthy controls. Although transcriptional changes in peripheral blood may not directly reflect enzyme expression or function in the CNS or gut, this downregulation positively correlates with ASD risk factors (i.e., parental age, iron, and vitamin D deficiency) and negatively with ASD scores [129]. A recent investigation of lymphoblastoid cell lines from 87 autistic individuals and 78 controls found that ASD patients exhibit reduced NADH production when Trp is the sole energy source. This data may suggest that Trp metabolism is altered, but cell lines are only a proxy and may not fully reflect brain-specific metabolic dynamics [130]. Such deficits may have implications for critical molecular processes during early brain development, particularly in the first month of gestation, mitochondrial homeostasis, and neuro-immune activity. Thus, an unbalanced production of Trp metabolites, including 5-HT, QA, and KYNA—which is exclusively observed in syndromic or non-syndromic autism—may contribute to abnormal neuronal network organization. This imbalance could disrupt the typical short- and long-range cortical pathways, as well as the excitatory/inhibitory ratio seen in ASD, potentially affecting even fetal cells [9, 131, 132].

The gut microbiome also plays a crucial role in ASD-related Trp metabolism. Dysbiosis in ASD patients may favor spore-forming bacteria (e.g., *Clostridium* sp.) [133], which are hypothesized to enhance peripheral 5-HT production via increased expression of tryptophan hydroxylase 1 (TPH1) and reduced MAO activity [133, 134]. These processes are notably active in both human and mouse neonates, which also show an increased abundance of 5-HT-producing bacteria [135]. Moreover, a reduction of bile-metabolizing *Bifidobacterium* and *Blautia* species in the gut of the BTBR T+ Itpr3tf/J mouse model of ASD is associated with deficient intestinal bile acid and Trp metabolism, marked GI dysfunction, and impaired social interactions. However, the BTBR model also displays high anxiety and altered stress responses, which complicate the interpretation of behavioral findings [136]. In the offspring of the maternal immune activation (MIA) mouse model, these ASD features have been shown to be ameliorated by oral treatment with the human commensal *Bacteroides fragilis *[89], which restores the increased indole pyruvate (IPy) characterizing MIA offspring to control levels*.* Additionally, higher levels of indole-3-acetic acid (IAA), which inhibits the mTOR pathway promoting the development of regulatory T (Treg) cells [137], and indolyl lactate [9]—both gut microbiota-derived Trp metabolites—characterize the urinary metabolome of ASD patients, while indole and 3-methylindole are increased in their feces [90].

Overall, ASD involves a significant Trp metabolism imbalance that may be influenced by the gut microbiota. Targeting intestinal microbes could help mitigate GI and behavioral symptoms.

### Attention Deficit Hyperactivity Disorder 

ASD and ADHD share overlapping risk genes and environmental influences, including prenatal and early postnatal factors. While ADHD has distinct features like impulsivity, both disorders exhibit traits such as executive dysfunction, hyperfocus, social challenges, sleep disturbances, and learning disabilities [138]. Similarly to ASD, Trp metabolism is a potential molecular mechanism underlying ADHD pathophysiology, as both conditions involve inflammation and immune dysregulation. Still, the effect of Trp metabolism on ADHD remains under-characterized compared to ASD [111, 116, 139, 140].

Early links between ADHD and the serotonergic system emerged from psychostimulants like Sydnocarb, which paradoxically calms ADHD models by increasing extracellular monoamines, including 5-HT [141]. It was later found that children with ADHD undergoing Sydnocarb treatment showed increased excretion of N-1-methylnicotinamide (N-MNA), along with a higher N-MNA/5-HIAA ratio, which may suggest (but does not directly demonstrate) a shift toward KP dominance over the 5-HT pathway. Given that Sydnocarb affects multiple monoamine systems, elevated N-MNA might alternatively reflect enhanced general monoamine turnover or oxidative stress [142].

A meta-analysis by Cavaleri et al. found that untreated ADHD patients consistently have elevated blood Trp and kynurenine levels but lower KYNA compared to controls [91, 92]. Similarly, a PTCHD1 knockout ADHD/ASD mouse model shows increased KYN, anthranilic acid, and 3-HK in serum and brain, along with hyperlocomotion, impulsivity, and impaired recognition memory [93]. ADHD patients also exhibit lower serum 3-HK levels and 3-HK/KYN ratios, which may disrupt cortical maturation, decrease NAD production [92, 94], and lower 3-hydroxyanthranilic acid (3-HAA) [95]. While these findings are promising, it remains unclear whether such metabolic changes are causal mechanisms, secondary effects, or adaptive responses.

Recent evidence indicates that methylphenidate, the most widely used first-line pharmacological treatment for ADHD, along with lisdexamfetamine [143, 144], modulates Trp metabolism. In ADHD patients, methylphenidate has been shown to significantly increase plasma levels of KYNA and xanthurenic acid [145]. In a subgroup of individuals with comorbid depressive symptoms (DS+), treatment also reduced anthranilic acid levels and urinary excretion of NADH and QA, normalizing these values to levels observed in non-depressed ADHD subjects and healthy controls [145]. Daily oscillations in indole-derived metabolites also appear to differ in DS+ ADHD subtypes. For example, hyperactive-impulsive DS+ individuals show elevated morning IAA levels, (IAA) which are halved following methylphenidate treatment. The drug also reduces elevated IPA levels and restores its normal diurnal variation [146]. Studies have reported that methylphenidate treatment alters gut microbiota composition, potentially linking changes in indole-related metabolites to microbial diversity in ADHD patients [147, 148].

These findings suggest that Trp metabolism may indicate treatment effects, offering potential biomarkers. Understanding how ADHD therapies affect all three Trp pathways (Fig. 1) may reveal new targets and aid in evaluating treatment response.

Despite these findings, research on Trp metabolism’s role in ADHD remains limited, highlighting the need for further studies.

### Rett Syndrome

Rett syndrome (RTT) is a rare X-linked genetic disorder caused by mutations in the *MECP2* (methyl CpG binding protein 2) gene. RTT is no longer listed under “neurodevelopmental disorders” in the DSM-5. However, it is still widely regarded in the scientific and clinical communities as a neurodevelopmental condition due to its early onset, developmental regression, and profound effects on brain maturation. Moreover, the ICD-11 classifies RTT under “Conditions with disorders of intellectual development as a relevant clinical feature”(LD90.4)*.*

While RTT shares clinical features and gut microbial traits with ASD, the role of Trp derivatives in the disorder is underexplored [149]. Early findings indicate that postmortem brains of RTT patients have increased KYN in specific areas, including the putamen, caudate nucleus, globus pallidus, raphe, and amygdaloid nucleus. 5-HT and 5-HIAA were decreased compared to healthy subjects [96]. Nonetheless, a study on 34 RTT patients found decreased plasma KYN levels and lower bacterial-derived Trp metabolites, indolepropionate and indolelactate—typically produced by *Clostridum sporogenes* species—compared to age- and gender-matched siblings. However, Trp was not altered in RTT subjects [98]. Peripheral reduction contrasts with the brain-specific increase in KYN, highlighting the need to understand regional Trp metabolism rather than relying on systemic measures.

In Mecp2 knockout mice, decreased 5-HT and serotonergic receptors (Htr2a, Htr3a) are linked to motor impairments [97]. In the RTT mouse brain, lower LAT1 transporter expression may limit Trp crossing the BBB, disrupting neural catabolic pathways [150]. Yet, whether this is a downstream effect of MeCP2 dysfunction or an independent co-pathology remains unclear. Moreover, the role of LAT1 in selectively limiting Trp and no other amino acids has not been directly validated in RTT models.

Alterations in Trp metabolism seem to be independent of specific Mecp2 mutations, with similar outcomes observed in mouse models of MeCP2 duplication syndrome, caused by an extra copy of the gene and characterized by intellectual disability and autistic-like phenotypes [99]. Metabolic profiles of this model showed significant changes in 5-HT levels, with increased serum 5-HT and decreased levels in urine and cecum compared to WT controls. Divergent 5-HT levels may reflect altered transport or compartmentalized metabolism rather than synthesis, highlighting the importance of metabolite dynamics over absolute levels. Finally, microbial profiling revealed an altered gut microbiome, with decreased *Firmicutes* (*Lachnospiraceae* spp., *Roseburia* spp., *Dorea* spp., and *Acetivibrio *spp.) and increased *Bacteroides* (*Bacteroides* spp. and *Parabacteroides* sp.). These shifts in microbial composition led to reduced levels of various indole and indole derivatives in plasma samples, including tryptamine, 4-hydroxyindole, 3-indolepropionic acid, indole-3-lactic acid (ILA), and 5-hydroxyindoleacetic acid [99]. Acting as AhR ligands, diminished levels of these indole derivatives could have consequences for multiple metabolic processes, including intestinal barrier integrity and inflammatory responses. However, in vivo functional studies directly linking decreased microbial indole production to RTT symptoms remain limited.

Despite these promising findings, research investigating the link between RTT and Trp metabolism is limited, warranting further investigation into its role in pathogenesis and comorbidities.

Summary of altered tryptophan (Trp) metabolites is reported in autism spectrum disorder (ASD), attention-deficit/hyperactivity disorder (ADHD), Rett syndrome (RTT), and MeCP2 duplication syndrome. For each metabolite, the direction of change (↑ or ↓), affected tissue, and evidence from preclinical and/or clinical studies are reported. Preclinical evidence refers to findings from animal models or in vitro systems, while clinical evidence includes studies involving biological samples from human patients. References correspond to those cited in the main text. The literature search was conducted on PubMed and Consensus (https://consensus.app/) using combinations of the keywords: “tryptophan metabolism,” “kynurenine,” “serotonin,” “indole,” AND each disorder name (e.g., “autism,” “ADHD,” “Rett syndrome,” “MeCP2 duplication”), with no time restriction and including both preclinical and clinical studies.

## Potential Therapeutic Strategies Targeting Tryptophan Metabolism in Neurodevelopmental Disorders

Trp metabolism is being investigated as a therapeutic target for cancer, neurodegenerative, and autoimmune diseases [22, 151–153]. Approaches include administering metabolites, using probiotics, and targeting key enzymes in 5-HT synthesis and KP (e.g., IDO1, TDO, and KMO) [154]. While most studies focus on modulating the 5-HT system, a few studies have applied therapeutic strategies targeting other Trp metabolic pathways to neurodevelopmental disorders.

Trp administration is used to modulate the serotonergic system [155], with prenatal Trp deficiency reducing serotonergic neurons by 35% [156] underscoring its importance for fetal neurodevelopment and prevention of neurodevelopmental disorders. While supported by preclinical data, human Trp levels are tightly regulated, and high-dose supplementation carries the risk of shunting toward potentially neurotoxic KP metabolites like QA [24, 157]. Defining an optimal Trp dose remains challenging due to variations in brain 5-HT levels across different regions and developmental stages. Additionally, factors such as limited patient availability in clinical trials, difficulties in matching control groups, and individual differences in diet, metabolism, and genetics further complicate standardizing Trp-based interventions.

Pharmacologically, targeting the 5-HT_1A_ receptor has shown promise in treating various symptoms associated with neurodevelopmental disorders [58], including respiratory deficits, anxiety, and stereotypical movements in ASD and RTT syndrome [158]. Indeed, treatment with the agonist 8-OH-DPAT improved social behavior and helped extinguish fear memory in an ASD rat model [159]. In line with this, low-dose buspirone, a 5-HT1A receptor agonist, has been proposed as an adjunct therapy for restrictive and repetitive behaviors in young children with ASD, alongside behavioral interventions [160]. Mirtazapine, a noradrenergic and serotonergic antidepressant, improves sleep and mood disturbances in both Mecp2+/− mice and RTT patients [161]. Selective serotonin reuptake inhibitors (SSRIs) like fluoxetine are commonly prescribed for ASD, with evidence supporting their benefits for psychiatric symptoms in RTT [162], and motor deficits in Mecp2 mouse models [163]. Clinical results, particularly in ASD, remain inconsistent, with SSRI efficacy varying with age, symptoms, and individual response. Side effects and the lack of stratified protocols currently limit their clinical application [164, 165].

Another potential therapeutic strategy involves modulating the enzyme tryptophan hydroxylase 2 (TPH2), which catalyzes the first and rate-limiting step in the biosynthesis of serotonin [166]. Recent research has resolved the cryo-EM structure of the TPH2 tetramer and identified ZINC000068568685 as a promising small-molecule activator with high binding affinity, laying the groundwork for novel TPH2-targeted drugs [167].

Metformin, a first-line treatment for type 2 diabetes, exhibits neuroprotective effects and may improve ASD behavioral phenotypes [168, 169]. Metformin has been shown to alter Trp, 5-HT, and 5-HIAA levels in the colon and cerebral cortex of high-fat diet-fed BTBR mice, a model of ASD. This may be due to its modulation of Trp metabolism, potentially restoring the 5-HT pathway, although its broad effects on the microbiome, insulin signaling, and mitochondria, obscure whether Trp modulation drives behavioral improvements [170]. Importantly, recent findings suggest that metformin reprograms Trp metabolism by directly influencing the gut microbiome in mice exposed to chronic stress, ultimately alleviating depressive-like behaviors [171].

Probiotic therapy targeting the gut-brain axis has also emerged as a promising intervention for ASD [172, 173]. Daily administration of *Lactobacillus helveticus* CCFM1076 in the VTA rat model of ASD has been reported to restore neurotransmitter homeostasis by improving the balance of the 5-HT system in the peripheral and CNS and ameliorating autistic-like behaviors [174]. Additionally, the probiotic *Bifidobacterium longum* CCFM077 has been reported to restore kynurenine metabolism in ASD rat models, improving autistic-like behaviors, while also regulating levels of QA, glutamic acid, and GABA, and reducing microglial activity in the cerebellum [175]. The QA level in the brain correlated with behavioral improvements, suggesting its potential as a biomarker for autism treatment. Pharmacological or dietary interventions targeting Trp pathways (e.g., enzyme inhibitors and probiotics) show promise in modulating neuroinflammation and neurotransmitter balance. While Trp metabolism represents a favorable therapeutic target for various pathological conditions [176], its application to neurodevelopmental disorders is still emerging and largely based on animal studies.

## Limitations of the Current Research Landscape

While growing evidence links Trp metabolism to neurodevelopmental disorders, several methodological limitations remain. Most findings come from animal models, postmortem tissue, or peripheral biospecimens, which may not reflect human CNS or developmental processes. Human studies are often cross-sectional, limiting causal insights, and rely on in vitro models or non-neural samples like urine or feces, raising concerns about compartment-specific relevance.

Many studies assess only a narrow set of metabolites, overlooking pathway complexity. Transcript-level data on key enzymes (e.g., TPH and IDO) often lack corresponding protein or activity measures. Inconsistencies in platforms, sample handling, and cohort variables (age, sex, diet, and microbiota) hinder comparability.

These limitations highlight the need for future research to adopt standardized, multimodal, and longitudinal designs to elucidate the developmental and clinical implications of disrupted Trp metabolism.

## Concluding Remarks and Open Questions

The existing literature underscores the complex interplay between multiple factors—maternal diet, stress exposure, gut health, intestinal microbiota and inflammatory responses—that influence one another and collectively shape Trp metabolism homeostasis. These factors have relevant implications for fetal brain development, with long-lasting effects on the offspring. However, the complexity of Trp metabolism, involving multiple interconnected biochemical pathways that compete for shared substrates and intermediates, continues to hinder a complete understanding of its physiological and pathological mechanisms. Several open questions remain: (i) How does the gut microbiota influence host Trp metabolism, limiting Trp availability for absorption, protein synthesis, serotonin, or KP metabolites? (ii) Beyond maternal stress, can postnatal stress alter peripheral and central Trp metabolic pathways, impacting brain network maturation and plasticity during critical periods of development? (iii) Can perinatal dysbiosis,such as prolonged antibiotic use or infant malnutrition disrupt Trp metabolism, triggering inflammatory responses and altering brain function? (iv) Does an early-life imbalance in the KP or gut microbiota-derived indole production contribute to the onset of ASD and ADHD, or exacerbate neurological symptoms in neurodevelopmental disorders more broadly? Although still in its early stage, research is revealing therapeutic potential. Substantial efforts are required to identify robust biomarkers and enable patient stratification based on metabolic profiles. Looking ahead, the discovery of specific metabolites or targetable molecules (i.e., enzymes and gut microbes) could pave the way for novel interventions. If applied during crucial developmental windows, perhaps beginning as early as the prenatal period, such strategies may counteract the long-term consequences of Trp metabolism disruption on brain function and neurological outcomes.

## Data Availability

No datasets were generated or analysed during the current study.
